# Renal Dropsy, Following Scarlatina

**Published:** 1873-02-01

**Authors:** N. S. Davis


### RENAL DROPSY, FOLLOWING SCARLATINA.

DELIVERED IN MEDICAL WARDS OF MERCY HOSPITAL, BY N. S. DAVIS, M.D.

GENTLEMEN:—There are but few general practitioners who have not found the sequelæ of scarlet fever among the most obstinate and serious ailments that come under their observation. Among these none are more important than the renal affections accompanied by anasarca.

We have before us to-day a case which well illustrates the important series of pathological phenomena developed in the progress of such cases. The patient, a girl aged fourteen years, usually in good health, was attacked with scarlatina simplex, on January 11th. The disease ran a very mild course, requiring but little medical treatment. By the 22d she seemed quite well again. When brought here on the 8th of February, we found her presenting the appearance of a moderate degree of general anasarca, with a dull pain in the loins and head, and a scantiness of urine, but no fever. She was directed to have two drachms of the bitartrate of potassa, and three drachms of the acetate, dissolved in half a pint of water, and to take a tablespoonful of the solution every four hours.

I did not see her again until the 13th. The prescription made on the 8th had appeared to have very little effect. The bloating of the whole surface had steadily increased, with increase of the pain in the head and back, frequent nausea, and some fever. The urinary secretion had decreased until the 12th, when it became entirely suppressed, and violent general convulsions began on the night following. The convulsive paroxysms followed each other in quick succession, allowing only an imperfect degree of consciousness to be recovered between them; the stomach promptly rejected all drinks by vomiting; the pulse was small and frequent; skin cool; pupils slightly dilated; respirations short, but regular, between the convulsive paroxysms. She had had no passage of urine or fæces during the preceding twenty-four hours. Immediately after the convulsions commenced, late in the evening of the 12th, the house physician gave the bromide and iodide of potassa freely, but without any perceptible effect. On my visit on the morning of the 13th, I ordered extensive warm fomentations, with a view of promoting the action of the skin, and gave internally a powder of hydrg. chlor. mite. 5 grs., and nitrate of potassa 5 grs., every two hours, with ten drops each of chloroform and fluid extract of cannabis Indica between. The latter was given more to prevent vomiting than for any supposed anti-spasmodic influence.

The convulsions continued to recur through the day, but at longer intervals ; otherwise there was no improvement in her symptoms, and no evacutions, either of urine or fæces. The further use of the powders was suspended, and in their place Croton oil, suspended in the form of an emulsion, was given, in doses of a little less than one drop every hour until evacuations should occur. When she had taken three drops, a part of which was rejected by vomiting, it began to operate freely upon the bowels.

On the morning of the 14th we found her quiet; no convulsions since the evening previous; face, and surface generally, still much bloated from dropsical infiltration; skin cool; pulse small, and 120 per minute; mind dull, but capable of being partially aroused to activity, and indications of partial paralysis of the left side.

The bowels had been evacuated freely several times, and she had passed a moderate quantity of urine twice. She was directed to have beef tea in small quantities for nourishment, and a solution of bitartrate of potassa for drink. On the 15th day she was much improved in all respects, except that the left arm was completely paralyzed, and the movements of the bowels had continued frequent, with some tenesmus, and mucus in the discharges. The urine was nearly natural in quantity and appearance. She complained of some pain in the paralyzed arm, between the shoulder and elbow.

The following prescription was ordered: ℞. Fl. ex. scutellaria, ℥ iii; tinct. digitalis, ℥ i; iod. potassa, ʒ iii. One teaspoonful to be taken every four hours. Also—℞. Ol. terebinth, ʒ iii; tinct. opii, ʒ ii; pulv. g. acacia, sacchar. alba, aa. ʒ iii. Rub together, and add spts. nitre, dulc., ℥ iss. aquæ mintha, ℥ iss. A teaspoonful every four hours, alternating with the other prescription, until the dysenteric irritation of the lower bowel ceases.

The use of the latter prescription was required only three or four times, and the patient improved steadily from day to day, until the anasarca, the paralysis, and nearly all symptoms of disease had disappeared. From the 18th to the 24th the patient continued cheerful and active in mind; appetite good; bowels regular; strength improving, and all the appearances of returning health. But there remained some anasarcous puffiness of the face and lower extremities, and the urinary secretion was unsteady. Some days it was natural in quantity and appearance, on others it was smaller in quantity, and turbid. She was confined to the use of light food, subjected to no injudicious exercise or exposure to atmospheric changes, and during the period last named, took for treatment only mild diuretics and tonics.

On the 24th, however, she again became dull, more anasarcous, and the stomach irritable, with only slight discharge of very high-colored urine; and before the next morning convulsions again came on as violent as in the first attack. The same means were resorted to, with the addition of a vapor bath, and the omission of the croton-oil, the powders of calomel and nitrate of potassa operating freely. The convulsions again ceased on the procurement of free intestinal evacuations and the return of renal secretions. But her subsequent progress to recovery has been very slow and vacillating, and is still imperfect.

Another somewhat similar case that I met with a few years ago was that of a girl aged twelve years, previously in good health, who was attacked with scarlatina simplex, at the same time with two or three other children in the same family, in one of whom it presented the anginose variety, which was the occasion of my being called in. At the time of my first visit the girl was sitting up, and complaining so little that the mother did not think she required medical treatment.

The rash had been well developed on the surface, the fever moderate, and the case free from any apparent complications. She convalesced, and appeared well for some ten days, when her face and limbs began to swell. I was then called again to see her, and found the surface generally pale, and swollen from anasarca; pulse moderately accelerated; skin dry, and but little above the natural temperature; head light or giddy; dull pain in the back, with sense of heaviness and lameness in bending the trunk on the pelvis; bowels inactive; stomach nauseated at times; appetite much impaired, and urine scanty and dark colored. On examination the latter was found to contain a few blood corpuscles, and much albumen, with epithelium and fibrinous shreds. She was directed to have a saline laxative to open the bowels, to be followed by a prescription containing digitalis, nitrous ether, and idoide potassa, to be taken every three hours.

Two days later when I saw her again, her bowels had moved very freely, and she had taken the medicine regularly, but there was no marked change in her symptoms. The urine had not increased in quantity and was more bloody.

She was ordered a solution of bitartrate of potassa, of which she was to take freely. The mixture of digitalis and nitrous ether was continued, a powder of potassa nit., hydrarg. chlor. mite, and pulv. doveri being added at night. Warm fomentations were also applied both to the loins and abdomen. The anasarca continued, however, slowly to increase; the pulse became smaller and more frequent; the head more dizzy; the stomach more irritable, and the urine more scanty, and more largely mixed with blood.

During two days she passed not more than three or four ounces in the twenty-four hours, and this more than half blood.

In the evening of the fourth day after my last visit, she was seized with several general convulsions. Living in a part of the city distant from my residence, a physiclan from the neighborhood was called in, who ordered a warm bath, followed by fomentations, and the liberal internal use of bromide and iodide of potass. The convulsions, however, continued to recur at short intervals, until I saw her on the next afternoon.

The urinary secretion had been entirely suppressed for the preceding twenty-four hours; the face and whole exterior surface of the body and limbs were much bloated; the pulse 130 per minute, small and weak; skin moist, and temperature nearly natural; pupils dilated, and mind incapable of being roused to consciousness. Warm applications to the trunk and lower extremities were continued, and the following prescription ordered: ℞. Hydrg. chlor. mite., 20 grs; potssa nitras, 30 grs. Mix, and divide into four powders; one to be taken every two hours until the bowels were freely moved; the operation to be aided by warm salt water enemas. To act as temporary antispasmodics, ten drops each of chloroform and fluid extract of cannabis Indica were given between the powders. After she had taken three of the powders and one or two enemas, the bowels began to move freely, and the convulsions ceased. Soon after, she also passed four or five ounces of turbid urine. During that night and the following morning she urinated three or four times, and the bowels were evacuated copiously, but without attention on her part. She remained quiet during the night, and at my visit at ten o'clock the following morning she could be aroused to partial consciousness, but was very feeble. She was ordered a solution of bitartrate of potassa and gum arabic for a drink, and a powder containing five grains of potassa nitras, and three grains of doveri, every three hours. All other remedies were dispensed with, except beef tea for nourishment.

From this time the urinary secretion continued to improve in quantity and quality; the skin continued moist; the pulse became slower and more full, and the mental faculties regained their activity. In about one week she had so far convalesced as to need no further attention.

In a practice extending over a period of more than thirty years, during which I have seen a fair proportion of scarlet fever patients, and have often seen some degree of renal dropsy as a sequel, the two foregoing are the first cases that have occurred in my own practice, of complete suppression of urine, and convulsions following this much-dreaded fever. Both these cases occurred after the mildest grade of fever, and in spite of some directly preventive treatment. And neither seemed to be benefited by any remedies except such as aided in the elimination of the retained elements of urine, by promoting the action of the skin, kidneys, and bowels.

